# Norepinephrine Inhibits Macrophage Migration by Decreasing CCR2 Expression

**DOI:** 10.1371/journal.pone.0069167

**Published:** 2013-07-02

**Authors:** Fangming Xiu, Mile Stanojcic, Marc G. Jeschke

**Affiliations:** Ross Tilley Burn Centre, Sunnybrook Health Science Centre, Sunnybrook Research Institute, Division of Plastic Surgery, Department of Surgery, Department of Immunology, University of Toronto, Toronto, Ontario, Canada; University of Medicine and Dentistry of New Jersey, United States of America

## Abstract

Increased incidences of infectious and septic complications during post-burn courses represent the main contributor to burn injury mortality. Sustained increases in catecholamine levels, especially norepinephrine (NE), contribute to immune disturbances in severely burned patients. The precise mechanisms underlying NE-mediated immunoregulation are not fully understood. Here we hypothesize that persistently elevated NE levels are associated with immunodysfunctions. We examined the effects of NE on the phenotype and functions of bone marrow-derived macrophages (BMMs). Whole mouse bone marrow cells were treated *in vitro* with 40 ng/mL of M-CSF and with 1 x 10^-6^ M or 1 x 10^-8^ M of NE or without NE for 7 days; cells were collected and stained with antibodies for CD11b, F4/80, MHC II and the inflammatory CC chemokine receptor 2 (CCR2). We found 1 x 10^-6^ M of NE inhibited MHC II and CCR2 expression on CD11b^+^/F4/80^+^ BMM cells. It also inhibited BMM proliferation by inhibiting CSF-1R expression. On the contrary, 1 x 10^-8^ M of NE slightly increased both MHC II and CCR2 expression on CD11b^+^/F4/80^+^ BMM cells but inhibited CD11b^+^/F4/80^+^ BMM proliferation. MCP-1 based migration assay showed that the migration of 1 x 10^-6^ M of NE-treated BMM toward MCP-1 was significantly decreased compared to BMM without NE treatment. Both 1 x 10^-8^ M and 1 x 10^-6^ M of NE enhanced TNF-α production and phagocytosis of FITC-Dextran. Intracellular staining of transcriptional factor MafB showed that 1 x 10^-6^ M of NE treatment enhanced its expression, whereas 1 x 10^-8^ M of NE decreased expression. Stimulation with LPS in the last 24-hours of BMM culture further decreased CCR2 and MHC II expression of these BMM, suggesting the synergistic effect of LPS and NE on macrophage. Our results demonstrate that NE regulates macrophage differentiation, proliferation and function, and may play a critical role in the dysfunctional immune response post-burn.

## Introduction

Despite major advances in the management of patient care following burn injury, the incidence of sepsis has significantly increased over the past two decades [1,2]. Burns covering more than 30% total burn surface area (TBSA) are associated with stress, inflammation, hypermetabolism and catabolism that lead to profound morbidity and mortality [3,4]. Burn injury is one of the most severe forms of trauma accompanied by stress responses. The stress response causes an immediate increase in catecholamines and in burn patients these levels can reach several folds, while persisting for prolonged periods [5,6]. Enhanced adrenergic stimulation and catecholamine release are important components of the pathophysiology of severe burn and sepsis. In an experimental model of burn sepsis, abrogation of bone marrow NE content with 6-OHDA resulted in a 62% survival rate compared to no survivors in NE-intact mice. Flow cytometric analysis of monocyte progenitors showed significantly more mature monocyte progenitors in NE-depleted mice [7], indicating that NE greatly influences monocyte maturation.

Phagocytes are an essential component of innate immunity and play an integral role in the immune response to burn injury. In severely burned and septic patients, myeloid commitment shifts toward monocytopoiesis [8,9] and dysfunctional macrophages (MΦ) are the hallmark of the disturbed immune response [10]. While a clear role norepinephrine plays in modulation of macrophages maturation [7,11], there have been controversial results of catecholamine-mediated alterations in phagocytosis and TNF production. One study of murine wound showed that both low (1 x 10^-9^ M) and high (1 x 10^-6^ M) doses of NE suppressed wound macrophage phagocytic efficiency [12]. However, studies of mouse peritoneal macrophages found that lower doses of NE enhanced phagocytosis and cytokine production, whereas higher doses of NE have less effectiveness [13,14]. Macrophages are major producers of pro-inflammatory mediators during burn injury and sepsis [10]. Increased release of pro-inflammatory factors by macrophage appears to be of fundamental importance to the development of post-burn immune dysfunction, particularly at the early stage of disease [10]. On one hand, studies have shown that NE enhances TNF-α secretion from macrophage [15,16], whereas others show NE inhibiting TNF-α secretion from splenic macrophages isolated both from a polymicrobial sepsis mouse model and wild type rats [17,18]. These conflicting results further emphasize the demand for further investigation.

After burn injury, immune cells (ie. monocytes and neutrophils) infiltration into the wounded area is an important step of the healing process. In this process, chemokine receptor-dependent migration toward chemokine gradient is essential. A recent study found that CX3CR1 deficiency resulted in decreased recruitment of CX3CR1-positive myeloid cells into the burn wound leading to decreased wound healing [19]. Another study found that CCR2 was important for neutrophil tissue infiltration during sepsis [20]. This chemokine receptor pathway may be an attractive therapeutic approach for wound healing [21], however the specific role played by CCR2 on macrophage in severe burn and sepsis is yet to be examined.

Taking into consideration that the effects of NE on macrophage function are not fully understood, the present study investigates the effects of NE on macrophage proliferation, maturation and function in a murine bone marrow *ex vivo* culturing system. We found that NE has a broad regulating effect on macrophage differentiation, maturation and activities such as phagocytosis, migration and cytokine secretion. Our findings provide new insights into the mechanisms by which the catecholamines modulate the immune response in severely traumatized patients.

## Materials and Methods

### Reagents and Abs

FITC-Dextran (MW: 40K) and LPS from *Escherichia coli* 0111:B4 were purchased from Sigma-Aldrich (Oakville, Ontario, Canada). Recombinant murine M-CSF was purchased from PeproTech (Rocky Hill, NJ, USA) and stock solution of 40 ng/mL was stored at -80°C. MCP-1 protein, RPMI 1640 lacking phenol red and charcoal-dextran-treated FBS were purchased from Invitrogen Life Technologies Inc. (Burlington, ON, Canada). BD Intracellular staining kit was from BD (Franklin Lakes, NJ, USA).

PE-conjugated rat anti-mouse CD11b, PerCPCy5.5-conjugated rat anti-mouse F4/80 and APC-conjugated rat anti-mouse CSF-1 receptor were purchased from Biolegend (San Diego, CA, USA). FITC-conjugated mouse anti-mouse I-Ab was from BD (Franklin Lakes, NJ USA). APC-conjugated rat anti-mouse CCR2 was purchased from R&D Systems (Minneapolis, MN, USA). FITC-conjugated TNF-α and rat IgG2b κ isotope control were from eBioscience (San Diego, CA, USA). MafB polyclonal primary antibody and FITC-conjugated goat anti-rabbit IgG were from Abcam Inc. (Toronto, ON, Canada).

### Animal and bone marrow-derived macrophage culture

All of the female C57BL/6 mice were purchased from Charles River Laboratories (St Constant, Quebec, Canada). All animals were housed maximum five per cage and maintained on a constant light: dark, 12:12 cycle. All animal procedures were approved by the Sunnybrook Health Science Center Animal Care Committee.

Murine bone marrow-derived macrophages were generated as previously described [22]. Bone marrow cells were prepared from femur and tibial bone marrow of C57BL/6 mice and the concentration was adjusted at 2 x 10^6^/mL. BMM cells were cultured in RPMI 1640 lacking phenol red supplemented with 40 ng/mL of mouse M-CSF, 2 mM glutamine, 100 U penicillin/0.1mg streptomycin/mL, 10 mM HEPES buffer, 10% of charcoal-dextran-treated FBS instead of regular FCS.

Total BM cells were seeded at 1 x 10^6^ cells per mL in 2 mL of media in a 24-well plate and treated with or without NE. Various doses of NE (final concentrations, 1 x 10^-8^ M or 1 x 10^-6^ M) were added at day 0. On day 3 and day 6, half of the culture media were removed and replaced with fresh hormone-deficient media containing 40 ng/mL of M-CSF. Macrophages were harvested on day 7. To activate macrophage, 50 ng/mL of LPS was added to the culture media at day 6 and cultured for another 24 hours. For the time point study, in addition to day 0, NE (final concentration, 1 x 10^-6^ M) was added on day 3 and day 6. To evaluate the effects of NE on macrophage number, cells were counted immediately upon the completion of the cell culturing.

### Cell proliferation assay

BMM proliferation regulated by NE was examined using the CyQUANT Cell Proliferation Assay Kit according to manufacturers instructions. Briefly, bone marrow cells were isolated as mentioned above and 2 x 10^5^ BM cells were seeded into each well of a 96-well plate and treated with various doses of NE (final concentrations, 1 x 10^-6^ M and 1 x 10^-8^ M) or without NE. On day 7, media were carefully removed, washed with PBS and plates were placed in a -80°C freezer. The standard curve was made using BMMs harvested from culture flask in accordance with the protocol. Based on the standard curve, cell numbers in each sample can be calculated.

### Cell staining and flow cytometry

After the seven day culture, cells were harvested after digestion with 10 x Trypsin/EDTA at 37°C for 25 min. To stain these cells, cells were incubated with Fc receptor blocker for 10 min at room temperature (RT) to block Fc receptors followed by phenotyping macrophage with antibodies for CD11b, F4/80, I-Ab, and CCR2 or CSF-1R in PBS containing 1% BSA. Labeled cells were all run on the BD LSR II Flow Cytometer and data was analyzed using FlowJo (v. 8.7) software.

### Phagocytosis assay

Phagocytosis of FITC-Dextran by macrophage was measured as the cellular uptake of FITC-dextran and quantified by Flow Cytometry. Bone marrow cells were isolated (as described in Generation of macrophage from BM cells) and treated with varying doses of NE (as described in Animal and bone marrow-derived macrophage culture). Treatment without any hormones served as a control. On day 7 of culturing, FITC-Dextran was added to the BMM culture at the final concentration of 200 μg/mL and incubated for 30 min at 37°C. Cells added with the same amount of FITC-Dextran and incubated at 4°C for 30 min were used as the baseline of macrophage phagocytosis. All cells were collected and stained with PerCPCy5.5-conjugated anti-F4/80 antibody and PE-conjugated anti-CD11b antibody. Internalization ability was evaluated at the percentage of FITC-positive cells gated on the CD11b^+^F4/80^+^ BMMs.

### Intracellular staining of BMM

Intracellular staining (ICS) of MafB was performed according to the direction of ICS kit (BD bioscience). Briefly, BMM cells were treated with NE (final concentrations, 1 x 10^-8^ M or 1 x 10^-6^ M). On day 7, the whole cells in each well were harvested. After Fc receptor blocking, cells were stained with antibodies for F4/80 and CD11b. To do intracellular staining, cells were first resuspended with 100 μL Fixation/Permeabilization solution for 30 min at 4°C. Next, fixed and permeabilized cells were stained with the MafB primary antibody for 30 min at 4°C. In the end, samples were stained with FITC-conjugated secondary antibody.

For intracellular cytokine staining (ICCS) of BMM, cells were cultured as before, LPS (final concentration, 50 ng/mL) and BD GolgiPlug (final concentration, 1μg/mL) were added in the last 4 hours of culture. After Fc receptor blocking, cells were stained with antibodies for F4/80 and CD11b. To do intracellular TNF-α staining, permeabilized cells were stained with antibody for TNF-α or its corresponding isotype control for 30 min at 4°C. TNF-α^+^ BMMs were calculated by gating on CD11b^+^F4/80^+^ BMMs. Data were collected with a BD LSR II Flow Cytometer and analyzed by FlowJo software.

### Migration assay

BM cells (3 x 10^5^) were seeded on a 24-well Millicell (8-μm pore size filter) in 300 μL media with 40 ng/mL M-CSF with NE (final concentrations, 1 x 10^-6^ M and 1 x 10^-8^ M) or without NE treatment. The lower chamber was occupied with 500 μL of the same media. At day 7, the inserts were transfered into a new 24 well plate. The lower chamber was added with RPMI 1640 medium with 1% hormone-deficient serum and MCP-1 (the final concentration, 100 ng/mL). Plates were incubated at 37°C for 4 hours. Cells in the lower chamber that passed through the filter were counted under a Carl Zeiss Primo Vert microscope (Carl Zeiss, Canada).

### Statistics

All comparisons were performed using one-way ANOVA analysis using GraphPad Prism version 4 (GraphPad Software, San Diego CA), with a p value less than 0.05 taken as statistically significant.

## Results

### NE regulates macrophage differentiation and maturation from bone marrow

**Figure 1:**
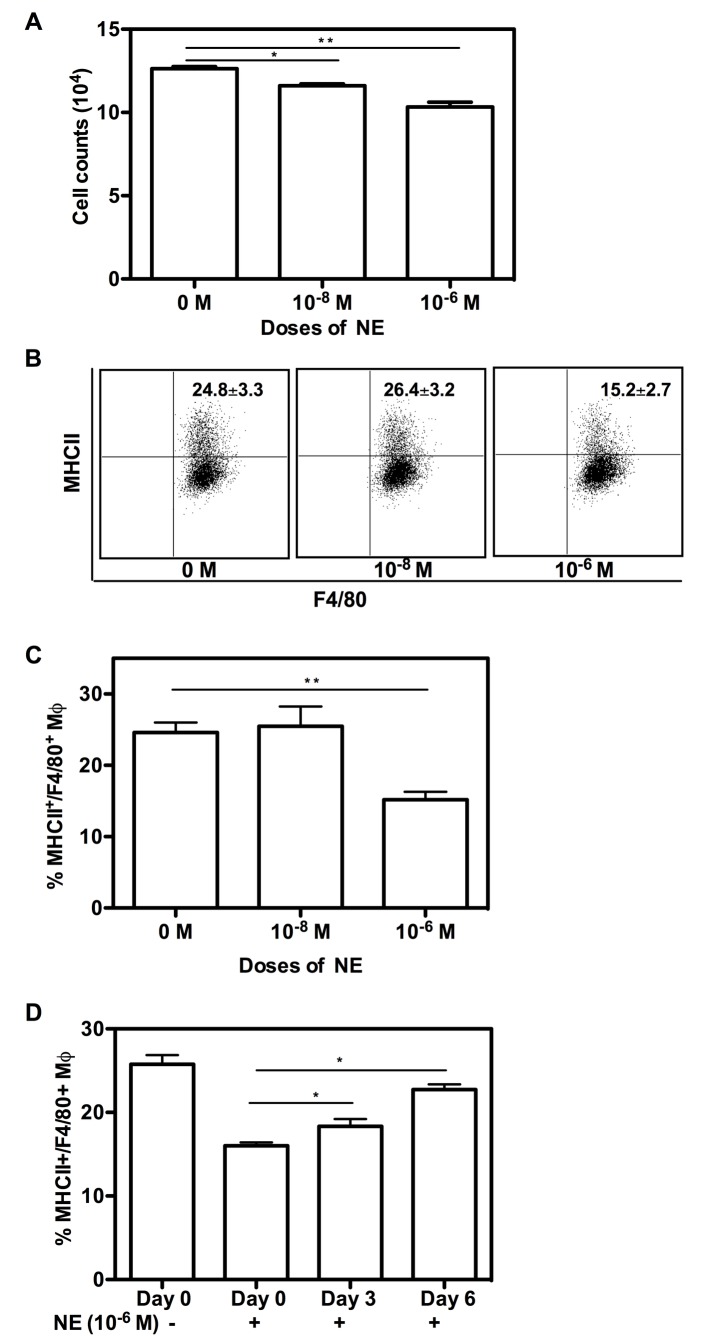
NE regulates macrophage proliferation and maturation from BM. Unfractionated BM cells were plated in a 24-well plate at 2 x 10^6^ cells/well and cultured for 7 days in hormone-deficient medium with murine M-CSF alone, or in varying concentrations of NE (1 x 10^-8^ M or 1 x 10^-6^ M) added at day 0, or in the presence of 1 x 10^-6^ M of NE added at different time points (day 0, 3 and 6). BMM proliferation was examined using the CyQUANT Cell Proliferation Assay Kit. Briefly, 2 x 10^5^ BM cells were seeded into each well of a 96-well plate and treated with various doses of NE or without NE. At day 7, cells were collected and total cell numbers were counted based on the standard curve (A). To phenotype BMM, cells stained with Abs for CD11b, MHC II and F4/80. Representative dot plot data of the percentage of MHC II^+^/F4/80^+^ Mφ are shown in (B). The graphic format of the percentage of MHC II^+^/F4/80^+^ Mφ is shown in (C). The time course of NE effects is shown in (D). Data show mean ± SD of 4 independent experiments. Significant difference is indicated as * p<0.05 or **p<0.01, compared to untreated control or different time points.

We established a very stable *ex vivo* BMM culture system. After seven days culture with 40 ng/mL of M-CSF, over 95% of cells were CD11b^+^/F4/80^+^ (Figure S1B). To examine whether NE can regulate macrophage proliferation and maturation from bone marrow, we cultured bone marrow cells for 7 days with or without the treatment of NE. First, we determined BMM proliferation by CyQUANT Cell Proliferation Assay (Figure 1A). Our results showed that 1 x 10^-8^ M and 1 x 10^-6^ M of NE significantly inhibited macrophage proliferation (p<0.05 and p<0.01, respectively).

We next examined the phenotype of the cultured BMM on day 7. Cells were harvested and stained with antibodies for macrophage markers such as CD11b and F4/80, as well as maturation marker MHC II. At the end of the culture, most of the cells harvested were CD11b^+^F4/80^+^ BMMs without NE treatment. As represented in Figure 1B and 1C, based on the percentage of MHC II^+^/F4/80^+^ BMM, 1 x 10^-6^ M of NE significantly inhibited MHC II expression compared to BMM without NE treatment (13.2 ± 2.7 vs. 24.8 ± 3.3%, respectively, p<0.01). Interestingly, 1 x 10^-8^ M of NE slightly increased the expression of MHC II, but it was not statistically significant.

To determine whether the presence of NE was required during the entire 7-day culture period to promote BMM proliferation, 1 x 10^-6^ M of NE was added to BM cells on different days after isolation (day 0, 3, or 6) and remained in culture until the cells were harvested on day 7. As shown in Figure 1D, 1 x 10^-6^ M of NE was most effective in inhibiting BMM maturation when added from day 0 of culture. The maturation-inhibiting effects of high doses of NE decreased daily as the culture progressed.

**Figure 2:**
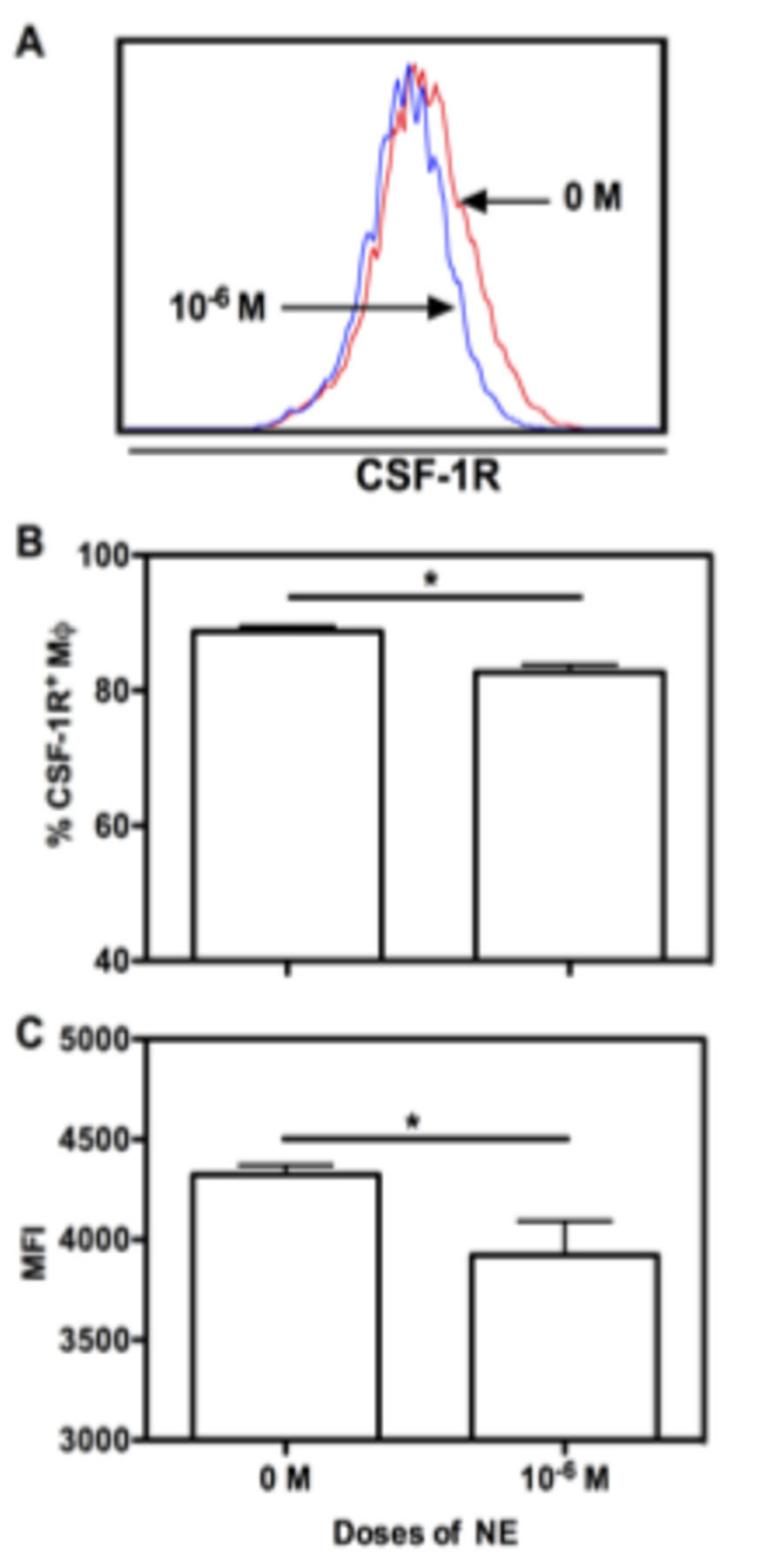
High dose of NE decreases CSF-1R expression. Unfractionated BM cells were cultured with murine M-CSF alone, or in the presence of varying concentrations of epinephrine. At day 7, cells were collected and stained with Abs for CD11b, F4/80 and CSF-1R. Based on the gating of CD11b^+^/F4/80^+^ BMM cells, representative histogram data of CSF-1R expression on Mφ is shown in (A). The corresponding graphic format data of CSF-1R expression on Mφ is shown in (B). Their corresponding MFI of CSF-1R expression on Mφ is shown in (C). Data show mean ± SD of 4 independent experiments. Significant difference is indicated as * p<0.05, compared to untreated control.

The inhibiting effects of NE on BMM proliferation may be attributed to decreased CSF-1R as shown in Figure 2. Compared to controls, both the percentage and MFI of CSF-1R^+^ BMM treated with 1 x 10^-6^ M of NE were higher (Figure 2A and B) (p<0.01 and p<0.05, respectively). We failed to detect an effect of low dose NE on CSF-1R expression (data not shown).

Taken together, our data showed the inhibiting effects of NE on BMM proliferation and maturation. High doses of NE (1 x 10^-6^ M) inhibited BMM proliferation and maturation, whereas low doses of NE (1 x 10^-8^ M) only slightly increased MHC II expression. Based on the time course studied, it is likely that NE acts on every differentiation stage of BMMs.

### NE inhibits macrophage migration by decreasing CCR2 expression

**Figure 3:**
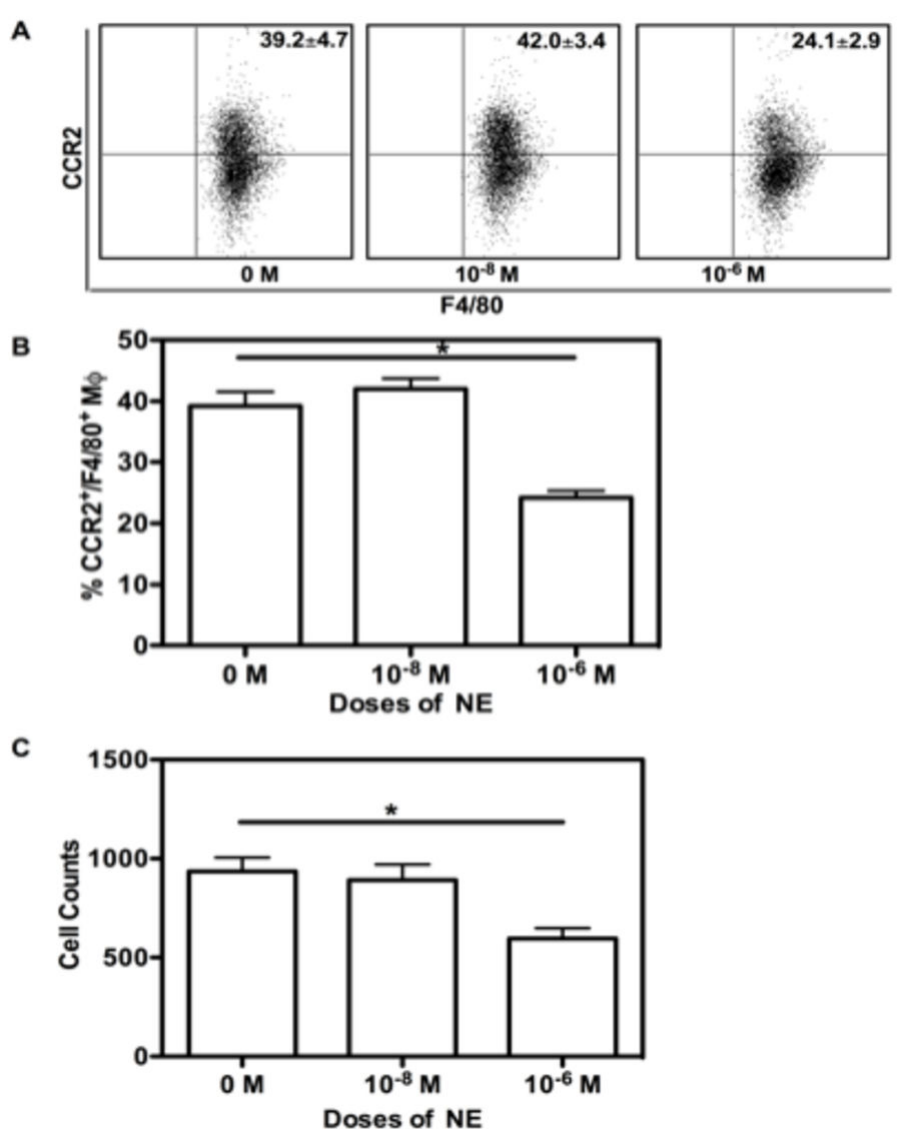
NE inhibits macrophage migration by decreasing CCR2 expression. BM cells were cultured in hormone-deficient medium with murine M-CSF alone, or in the presence of varying concentrations of NE. At day 7, cells were collected and stained with Abs for CD11b, F4/80 and CCR2. Based on the gating of CD11b^+^/F4/80^+^ cells, representative dot plot for percentage of CCR2^+^/F4/80^+^ Mφ is shown in (A). The corresponding graphic format data of percentage of CCR2^+^/F4/80^+^ Mφ is shown in (B). The comparison of cells that passed through insert filter after 4 hours incubation with MCP-1 (final concentration, 50 ng/mL) in the lower chamber are shown in Figure 2 (C). Data show mean ± SD of 4 independent experiments. Significant difference is indicated as * p<0.05, compared to untreated control.

Chemokine receptors such as CCR2 are very important for monocyte/macrophage egressing from bone marrow to circulation and migrating into tissues during chronic inflammation or acute infection [23,24]. Thus, we determined the CCR2 expression of NE treated bone marrow-derived macrophages. As seen in Figure 3A and 3B, 1 x 10^-6^ M of NE significantly decreased CCR2 expression based on the percentage of CCR2^+^/F4/80^+^ macrophages compared to controls (39.2 ± 4.7 vs. 24.1 ± 2.9%, p<0.05). However, 1 x 10^-8^ M of NE did not have significant effects on CCR2 expression.

To explore whether the decrease in CCR2 by 1 x 10^-6^ M of NE could lead to inhibited migration towards MCP-1, we utilizing the Transwell Migration Assay. After a 4 hour incubation, cells that migrated to the lower chamber were counted. As shown in Figure 3C, 1 x 10^-6^ M of NE treated BMM had significantly less cells compared to those without treatment or with 1 x 10^-8^ M of NE. Although 1 x 10^-8^ M of NE slightly increased CCR2 expression, it did not lead to altered migration. Therefore, only a high dose of NE (1 x 10^-6^ M) has significant effects on CCR2 expression and migration of BMM.

### LPS exacerbates NE’s effects

**Figure 4:**
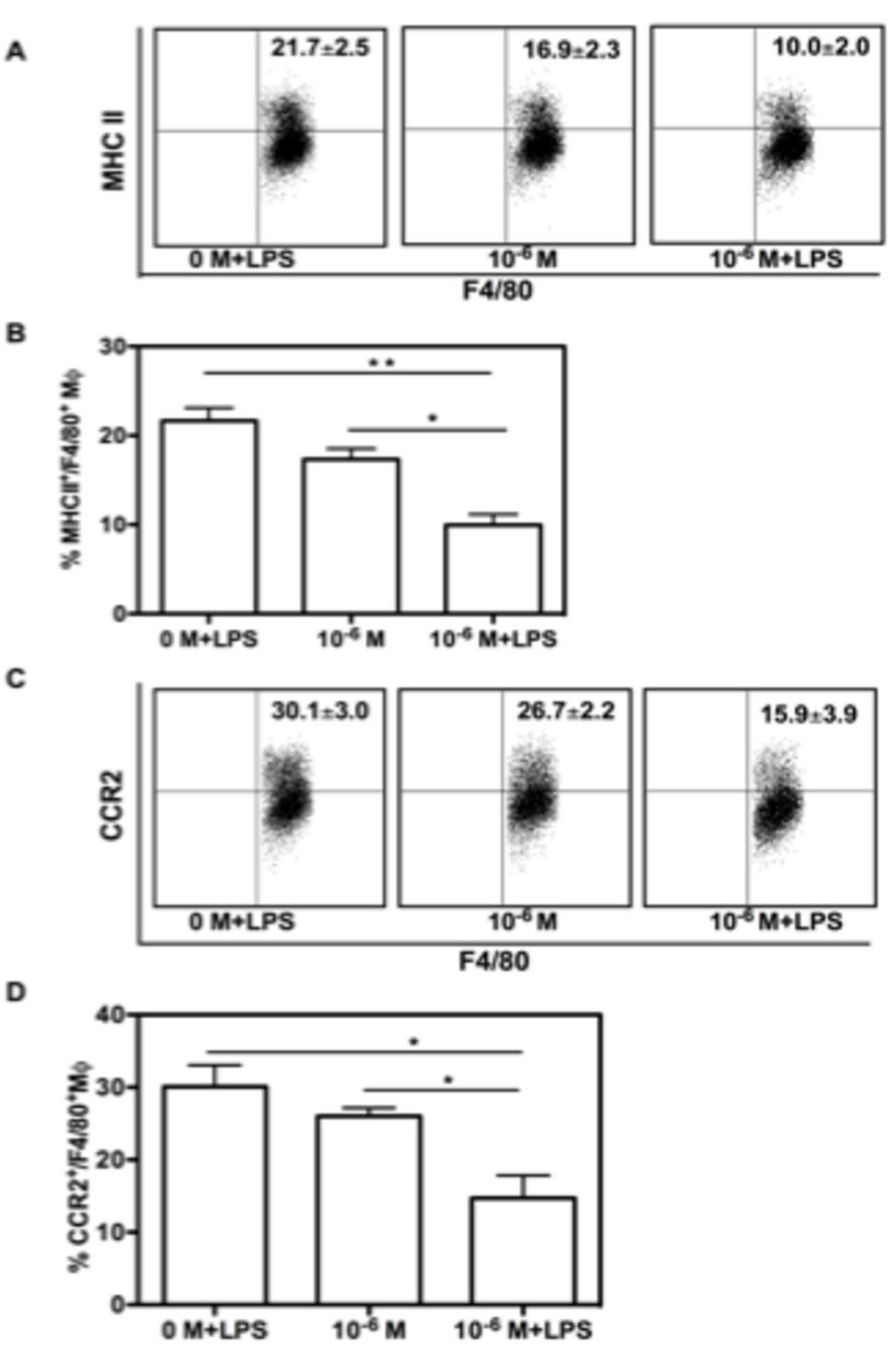
LPS exacerbates NE’s effects. In the last 24 hours of 7 day culture of BM cells with the treatment of NE, LPS (50 ng/mL) was added to the culture. At the end of culture, cells were collected and stained with Abs for CD11b, MHC II and CCR2 antibodies. Representative dot plot data and their corresponding graphic format data of percentage of MHC II^+^/F4/80^+^ Mφ are shown in (A) and (B) respectively. Representative dot plot data and their correspondent graphic format data of percentage of CCR2^+^/F4/80^+^ Mφ shown in (C) and (D) respectively. Data show mean ± SD of 4 independent experiments. Significant difference is indicated as * p<0.05 and ** p<0.01, compared to untreated control.

It has been well documented that LPS down-regulates the CCR2 expression of macrophages [25,26]. Taking into consideration the known elevation in LPS in severely burned patients due to disrupted intestinal permeability [27], thus we explored whether LPS and high dose of NE (1 x 10^-6^ M) have combinational effects. LPS (50 ng/mL) was added to the BMM culture (1 x 10^-6^ M or 0 M) at day 6 and cultured for 24 hours. We found that LPS had additional effects with high dose NE (1 x 10^-6^ M) including BMM maturation and CCR2 expression. The percentage of MHC II^+^/F4/80^+^ MΦ in LPS alone and 1 x 10^-6^ M NE alone were 21.7 ± 2.5 and 16.9 ± 2.3, respectively, whereas it was 10.0 ± 2.0 in LPS with 1 x 10^-6^ M of NE (Figure 4A). The differences between the combination of 1 x 10^-6^ M of NE alone, LPS alone or both combined were statistically significant (Figure 4B) (p<0.05 and p<0.01, respectively). Similarly, compared to treatment with either LPS alone or NE alone, treatment with 1 x 10^-6^ M of NE plus LPS induced significantly lower expression of CCR2 in BMM (Figure 4C and D) (30.1 ± 3.0 vs. 15.9 ± 3.9, 26.7 ± 2.2 vs. 15.9 ± 3.9, respectively). Taken together, our results demonstrated that LPS exacerbated NE’s effect on the expression of MHC II and CCR2 on BMMs.

### NE promotes macrophage phagocytosis

**Figure 5:**
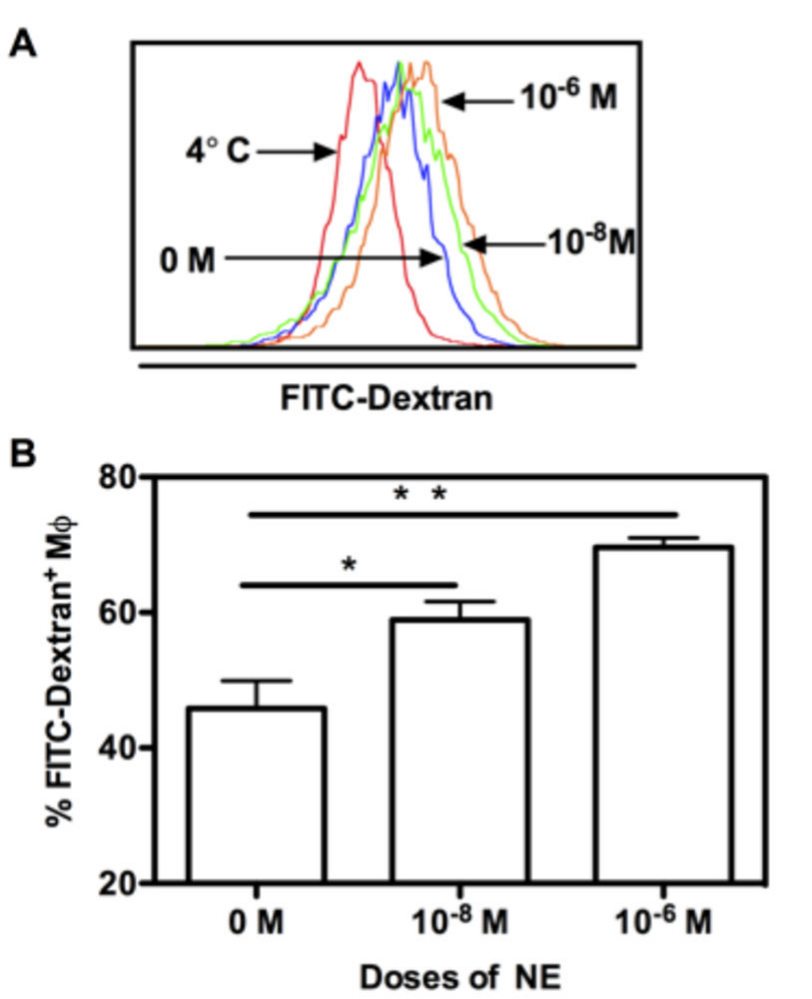
NE regulates antigen uptake by BMM. The whole bone marrow cells from C57BL/C mice were cultured with M-CSF in the presence of NE (1 x 10^-8^ M and 1 x 10^-6^ M) for 7 days. At day 7, FITC-Dextran (200µg/mL) was added to the culture for further 30 min culture at 37°C. As a negative control, FITC-Dextran-added wells were incubated at 4°C for 30 min. At end of the experiment, cells were collected and stained with antibodies for CD11b and F4/80. The percentage of FITC-Dextran-positive CD11b^+^/F4/80^+^ BMM was obtained by gating on CD11b^+^/F4/80^+^ BMM. Representative flow cytometric data are shown in (A) and corresponding graphic data are shown in (B). Bars represent FITC-Dextran-positive DCs as the mean of 4 independent experiments ± SD. *p<0.05; ** p<0.01, compared to untreated control (0 M).

To examine whether BMM treated with NE had altered phagocytosis, FITC-Dextran was added to the BMM culture on day 7 for 30 minutes and FITC-Dextran^+^ BMM were determined by FACs. As shown in Figure 5A and B, both high and low doses of NE enhanced BMM phagocytosis of Dextran compared to treatment with 0 M of NE, in a dose-dependent manner. As the baseline control, the percentage of FITC-Dextran^+^ BMM at 4°C was only 11% (data not shown). At 0 M, their were 42.8% Dextran^+^ BMM, whereas their were 58.9% and 69.6% for 1 x 10^-8^ M and 1 x 10^-6^ M, respectively. The differences in the proportion of FITC-Dextran^+^ BMM between 0 M and 1 x 10^-8^ M along with 1 x 10^-6^ M were statistically significant (p<0.05 and p<0.01, respectively). Our results demonstrated that both 1 x 10^-8^ M and 1 x 10^-6^ M NE enhanced the phagocytosis of BMMs in a dose-dependent manner.

### NE enhance TNF-α production

**Figure 6:**
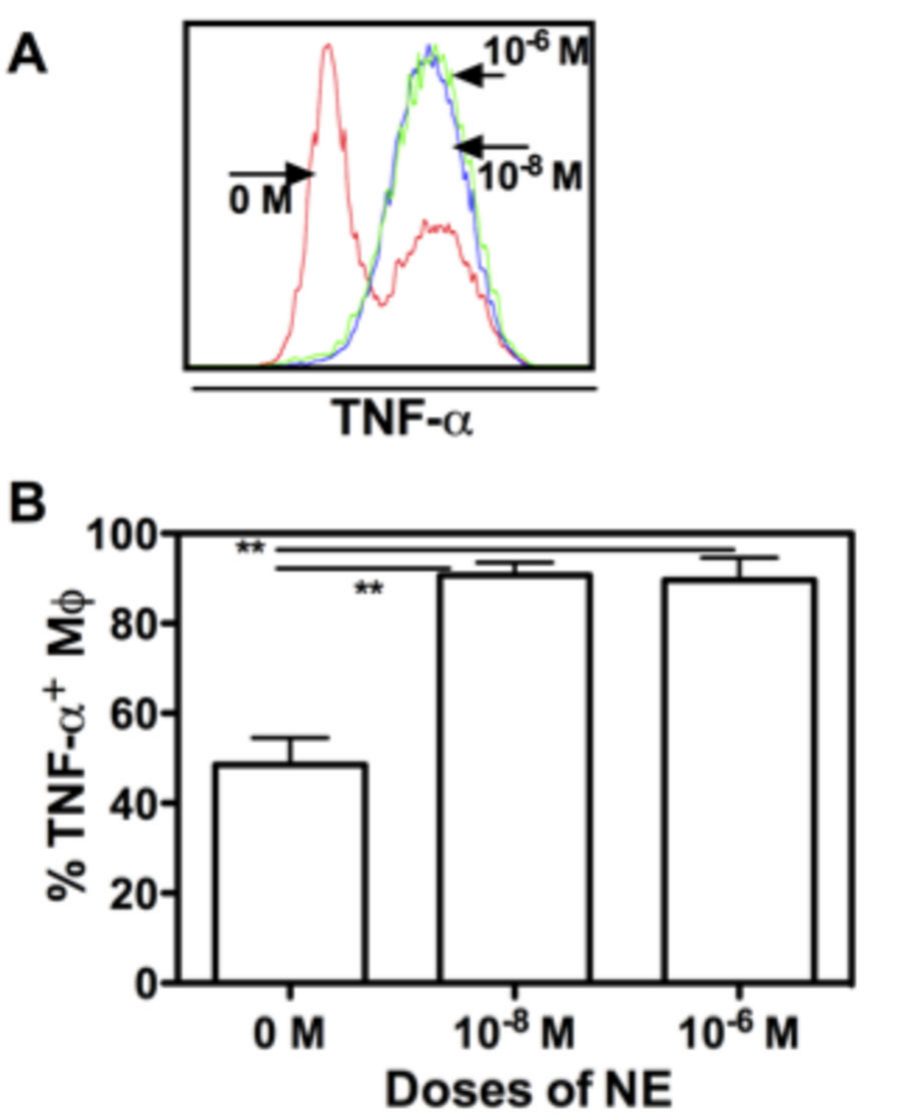
NE enhances TNF-α production from BMM. Bone marrow cells were cultured with M-CSF in the presence of NE (1 x 10^-8^ M and 1 x 10^-6^ M). At day 7, LPS (50 ng/mL) and the protein transport inhibitor GolgiPlug (final concentration, 1 µg/mL) were added to the culture for further 4 hour culture and then cells were collected and sequentially stained with membrane antibodies for CD11b and F4/80, and intracellular antibody of TNF-α. Representative flow cytometric data was shown in (A) and corresponding graphic data in (B). Graph bars represent TNF-α^+^ BMM as the means ± SD of 2 independent experiments in triplicate each time. ** p<0.01, compared to untreated control (0 M).

Macrophages are a major source of many cytokines involved in immune response in burn and sepsis [10]. To examine whether NE had an effect on BMM cytokine secretion, we determined TNF-α secretion after LPS stimulation for 4 hours. As represented in Figure 6 A and B, without NE treatment, there was only 45% of CD11b^+^/F4/80^+^ BMMs secreting TNF-α. However, over 90% of NE-treated BMM secreted TNF-α. The effects of NE peaked at 1 x 10^-8^ M. A concentration of 1 x 10^-6^ M did not further increase the percentage of TNF-α^+^ BMM cells. Taken together, these findings suggest that adrenergic stimulation may influence peripheral tissue macrophage inflammatory cytokine response following trauma and sepsis.

### NE regulates MafB expression

**Figure 7:**
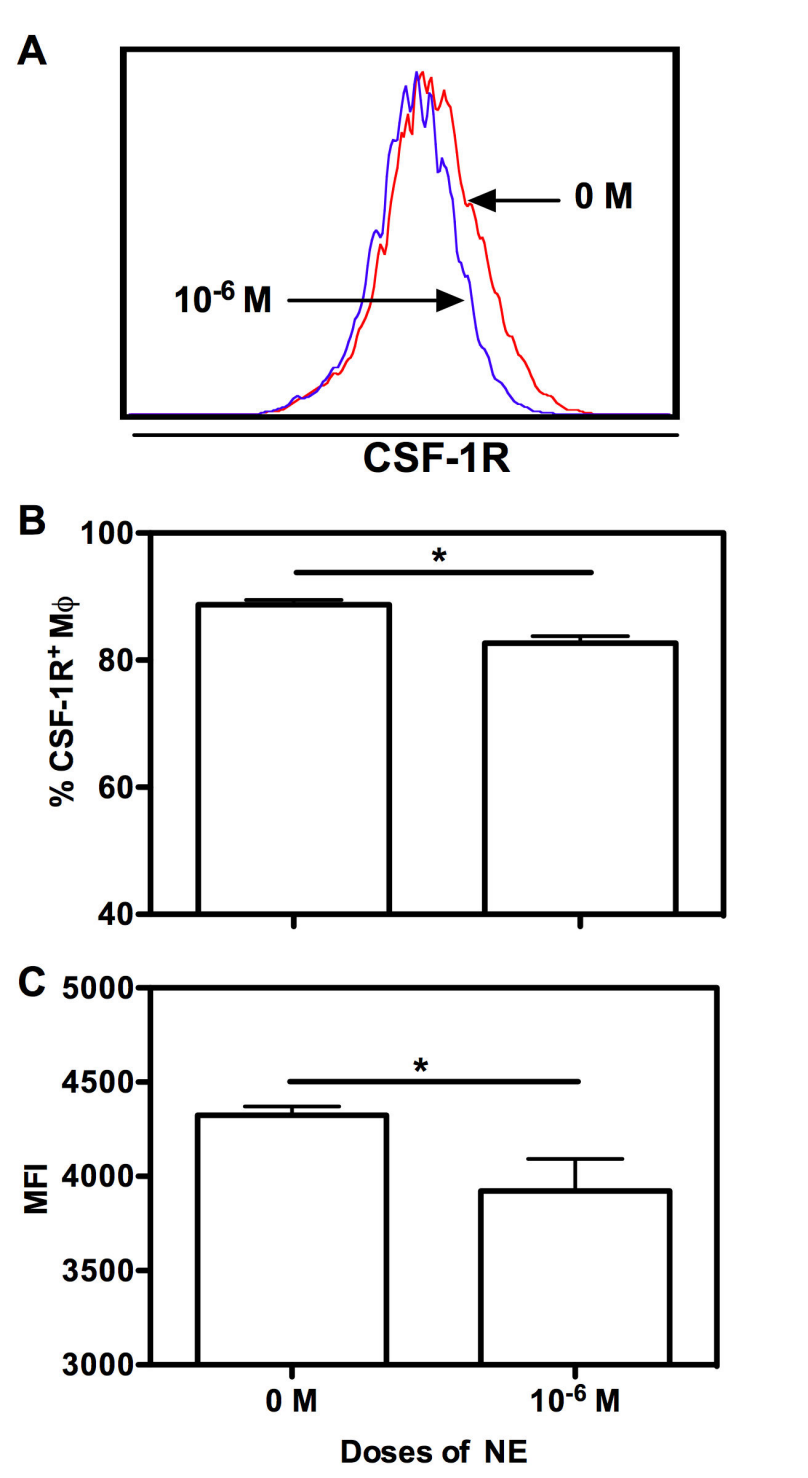
NE regulates MafB expression of BMM. At the end of the 7 day BMM culture, cells were harvested and stained with antibodies for phenotypic markers CD11b and F4/80 followed by sequential intracellular staining with primary MafB antibody and FITC-conjugated secondary antibodies. Representative dot plot data of percentage of MafB^+^ BMM were shown in (A) and their corresponding graphic format in (B). The MFI of MafB^+^ BMM were shown in (C). Data represent samples in triplicate for each time of two independent determinations. * p<0.05, ** p<0.01, compared to non-hormone treated control (0 M).

To investigate the mechanisms of NE’s effects on BMM differentiation, proliferation and maturation, we determined the expression of MafB, one of the essential transcriptional factors for macrophage differentiation [28], in BMM cells. As shown in Figure 7A, without NE treatment, only 71.8 ± 4.6% of BMM expressed MafB, whereas 1 x 10^-8^ M and 1 x 10^-6^ M groups were 55.3 ± 3.3 and 97.5 ± 3.1%, respectively. The difference between the control and the treatment with 1 x 10^-8^ M or 1 x 10^-6^ M were statistically significant (p<0.05 and p<0.01, respectively). The MFI of MafB^+^ BMM is the measure of MafB expression per cell. Compared to BMM without NE treatment, MFI of MafB^+^ BMM treated with 1 x 10^-8^ M of NE or with 1 x 10^-6^ of NE was significantly altered (Figure 7C) (p<0.05 and p<0.01, respectively). Since enhanced expression of MafB limits myeloid progenitor proliferation and accelerate macrophage differentiation [29], increased expression of MafB in 1 x 10^-6^ M of NE-treated BMM will push macrophage progenitors to mature quickly to macrophage, which leads to inhibited cell number as shown in Figure 1A. Overall, our results suggest that NE may regulate macrophage differentiation, proliferation and maturation by regulating MafB expression.

## Discussion

Dysfunctional macrophage is one of the hallmarks of severe burn and sepsis [10]. However, the mechanism underlying macrophage dysfunction during severe trauma and illness are not fully understood. In the present study, we explored the effects of NE on macrophage differentiation, maturation and function. Using the *ex vivo* bone marrow-derived macrophage culturing system, our study demonstrates that NE has comprehensive regulatory effects on macrophage differentiation, maturation and function. First, we showed that NE regulates BMM proliferation and maturation. Both high and low doses of NE inhibit BMM proliferation. However, only low dose of NE enhances BMM maturation as determined by MHC II and F4/80 expression. Secondly, high dose of NE (1 x 10^-6^ M) regulates macrophage migration by decreasing CCR2 expression. The migration of macrophages from circulation into tissue plays an essential role for wound healing. Our data suggest that NE may regulate tissue immune response by regulating CCR2-dependent monocyte tissue infiltration in severe burn and sepsis. We also showed that both 1 x 10^-8^ M and 1 x 10^-6^ M of NE enhanced macrophage phagocytosis. Since increased macrophage phagocytosis is essential for a faster wound healing, NE may promote wound healing in this regard. Fourth, both high and low doses of NE enhanced TNF-α production. Macrophages are major producers of pro-inflammatory mediators following thermal injury and hyperactivity is of critical importance to the development of post-burn immune dysfunction, such as systemic inflammatory response syndrome (SIRS) [10]. Our results show that concentrations as low as 1 x 10^-8^ M NE significantly increase TNF-α production from BMM, under the stimulation of LPS. Furthermore, it indicates that catecholamines play a role in acute inflammation during burn. We also found that epinephrine has similar effects on macrophage but with a less efficient mode (Figure S2). In summary, our study suggests that catecholamines may play a significant role in disturbed immune response during severe burn and sepsis by regulating macrophage differentiation and function.

The modulatory effects of NE on macrophage function are particularly prominent in phagocytosis and cytokine secretion. For macrophage phagocytosis, our results are in contrast to previous studies [12,30]. One of those studies using macrophage isolated from burn wound showed that NE inhibited macrophage phagocytosis of *Escherichia coli* [12]. This discrepancy may be due to the activation status and source of macrophage used in the experiments. A study on the effects of stress on macrophage behaviours found that stress caused a decrease in phagocytosis mediated by Fc-γ or mannose receptors in resting peritoneal macrophage, whereas increased phagocytosis in peritoneal macrophage isolated from mice that had been intraperitoneally injected with LPS 4 days before the experiment [31]. The increased cytokine secretion presently shown in our study is consistent with studies using thioglycollate-elicited mouse peritoneal macrophages [16], whereas other reports of NE modulation on cytokine function show decreases using normal rat spleen and CLP splenic macrophage [17,18]. Though we failed to show the dose-dependent effect of NE on macrophage TNF-α production in our system, other researchers showed the difference between different doses. It was reported that low levels of NE result in enhanced production of TNF-α [15,16], whereas high levels of NE inhibit TNF-α production [15,17]. Therefore, the effect of NE on macrophage activity is largely dependent on the activation status and source of macrophage as well the doses of NE.

LPS or endotoxin is an important structural component of the outer membrane of Gram-negative bacteria and is one of the best studied among pathogen-associated molecular pattern of bacteria. Nearly three decades ago, Maejima et al. showed that serious injury led to translocation of enteric bacteria into the mesenteric lymph nodes and liver because of gut barrier disruption [27]. Dr. Mannick’s laboratory further extended the notion that circulating LPS triggered the systemic inflammatory response syndrome in severe trauma and burn injury [32]. LPS has been shown to decrease CCR2 expression of macrophage [25,33], however the combinatory effects of LPS and catecholamines on macrophage function have not yet been studied. Our results demonstrated that LPS exacerbates NE’s effects on macrophage maturation and CCR2 expression. Therefore, in severe trauma and sepsis, when both LPS and catecholamine are increased, collective exposure will worsen the microenvironment for monocyte progenitors differentiation and maturation. In addition, macrophage activity can be sensitized by endotoxin. Assessing the effect of burn insult on endotoxin shock, Enomoto et al. found that endotoxin released from permeabilized intestine exacerbates burn outcome by sensitizing Kupffer cells [34,35]. Our own results showed that in the presence of LPS, NE’s effect peaks at 1 x 10^-8^ M, Further increases in NE dose did not enhance the production of TNF-α. Taken together, this suggests that the interactions between LPS and catecholamine are essential to regulating macrophage activities during severe trauma.

Macrophage is an important member of the mononuclear phagocyte system (MPS), which includes monocyte, macrophage and DC. Cells in the bone marrow that are committed to MPS are the macrophage and DC progenitor (MDP). Lineage commitment and differentiation of bone marrow progenitors toward MPS is tightly orchestrated by specific transcription factors and essential cytokines [36] and this is dysregulated in sepsis [37–40]. The pool of Lin- Sca-1+ c-kit+ (LSK) cells, which are enriched in bone marrow hematopoietic stem cells, is expanded in a sepsis model [37]. Although it has not been known whether bone marrow hematopoietic stem cells (HSC) sense infection directly, it does respond to LPS [39]. Both LPS signaling and reduction in bone marrow cellularity alone can induce the expansion of bone marrow hematopoietic stem and progenitor cells [37,38]. Our results suggest that catecholamine may act directly on macrophage progenitors since 1 x 10^-6^ M of NE was most effective in inhibiting BMM maturation when added from day 0 of culture (Figure 1D). Due to the higher expression of transcription factors like MafB and receptor for M-CSF, GMPs is skewed to favor monocyte commitment [40] [8]. In contrast to their results, our data showed that higher expression of MafB (Figure 7) and decreased expression of CSF-1R (Figure 2) on high dose of NE-treated BMMs contribute to inhibited differentiation and proliferation of BMMs. The emergency myelopoietic response to severe trauma redirects progenitor differentiation in severely burned and septic patients, but the differentiation toward macrophage and DC fails in time for chronically inflamed patients.

In conclusion, our findings on the effects of catecholamines on macrophage differentiation and function are important. This study was the first to show that catecholamines regulate CCR2 expression in BMMs. Our results not only provide greater insight towards understanding the pathophysiology of severe burn and sepsis, but also raise some concerns regarding immunotherapies targeting CCR2 in septic patients. A recent report found that CCR2 is essential for neutrophil infiltration during sepsis and suggested that targeting CCR2 might be a novel immunotherapy for sepsis [21]. However, our results present some inherited challenges. Due to the dual role of NE on CCR2 expression in macrophages, caution should be taken when targeting CCR2 in sepsis. Future studies should extend the current findings and examine CCR2 expression on monocytes/macrophages in an animal model or clinical patients.

## Supporting Information

Figure S1 **Gating schemes**.
Unfractionated BM cells were plated in a 24-well plate at 2 x 10^6^ cells/well and cultured for 7 days in hormone-deficient medium with murine M-CSF alone. At day 7, cells were collected and stained with Abs for CD11b and F4/80. Representative SSC/FSC is shown in (A) and the percentage of CD11b^+^/F4/80^+^ Mφ in the culture without NE treatment is shown in (B).
(TIF)

Figure S2 **Epinephrine regulates MHC II and CCR2 expression of BMM.**
Unfractionated BM cells were plated in a 24-well plate at 2 x 10^6^ cells/well and cultured for 7 days in hormone-deficient medium with murine M-CSF alone, or in varying concentrations of epinephrine (1 x 10^-7^ M or 1 x 10^-5^ M) added at day 0. At day 7, cells were collected and stained with Abs for CD11b, MHC II, CCR2 and F4/80. Representative dot plot data of the percentage of MHC II^+^/F4/80^+^ Mφ and CCR2^+^/F4/80^+^ Mφ are shown in (A) and (C), respectively. The graphic format data of the percentage of MHC II^+^/F4/80^+^ Mφ and CCR2^+^/F4/80^+^ Mφ are shown in (B) and (D), respectively. Data show mean ± SD of 4 independent experiments. Significant difference is indicated as * p<0.05, compared to untreated control.
(TIF)
