# Correction: Norepinephrine Inhibits Macrophage Migration by Decreasing CCR2 Expression

**DOI:** 10.1371/annotation/fc696ecf-aabf-411b-9819-238fd014d23c

**Published:** 2014-01-03

**Authors:** Fangming Xiu, Mile Stanojcic, Marc G. Jeschke

Figure 7 is incorrect. The corrected Figure 7 can be found here: http://www.plosone.org/corrections/pone.0069167.cn.g007.tif

