# Supplementary figures and images for: Norepinephrine Inhibits Macrophage Migration by Decreasing CCR2 Expression
Source: PLoS One. 2013 Jul 2;8(7):e69167. doi: 10.1371/journal.pone.0069167 (PMC3699643; doi:10.1371/journal.pone.0069167)

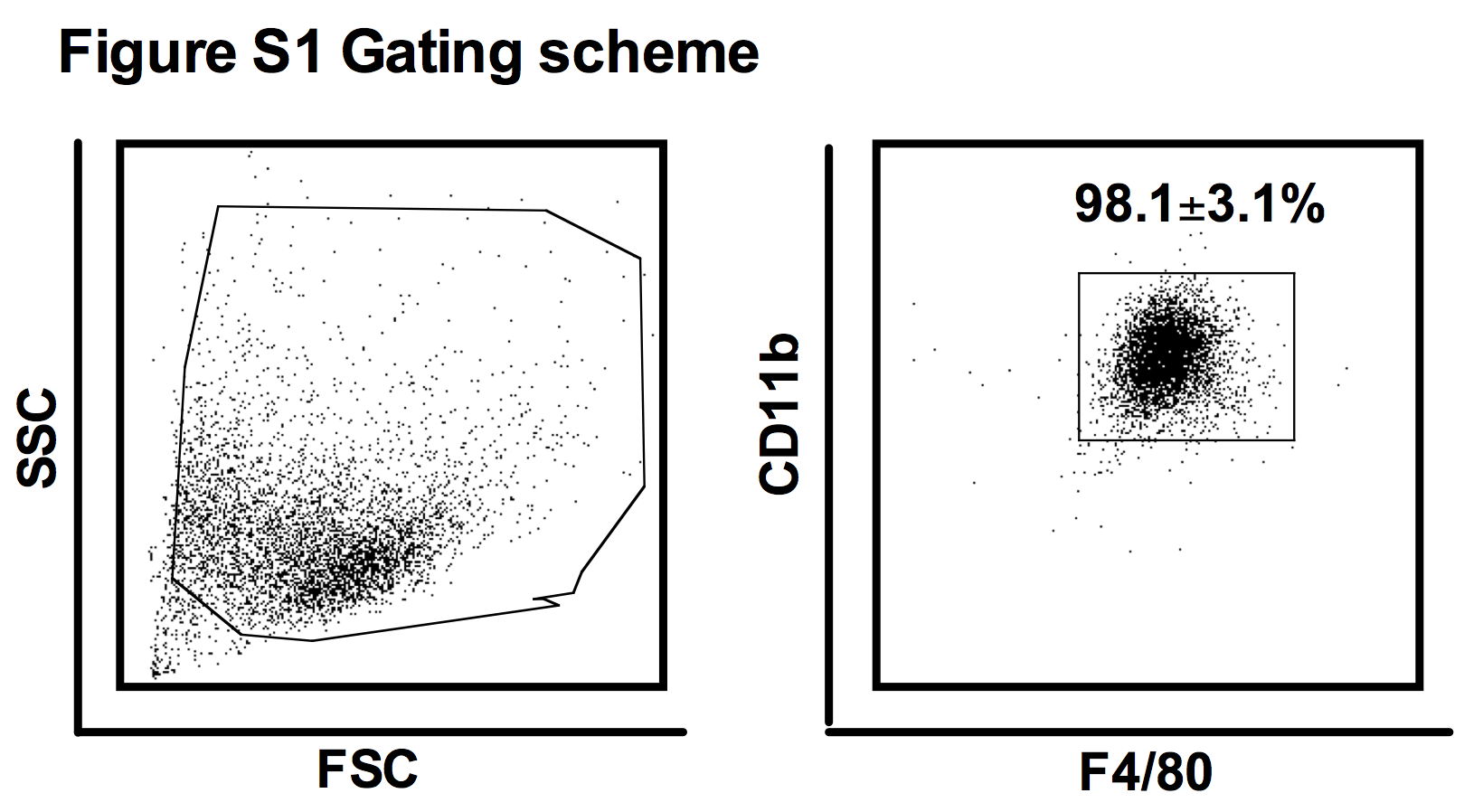

Supplement: Figure S1 — Gating schemes. Unfractionated BM cells were plated in a 24-well plate at 2 x 10^6^ cells/well and cultured for 7 days in hormone-deficient medium with murine M-CSF alone. At day 7, cells were collected and stained with Abs for CD11b and F4/80. Representative SSC/FSC is shown in (A) and the percentage of CD11b+/F4/80+ Mφ in the culture without NE treatment is shown in (B). (TIF) [file pone.0069167.s001.tif]

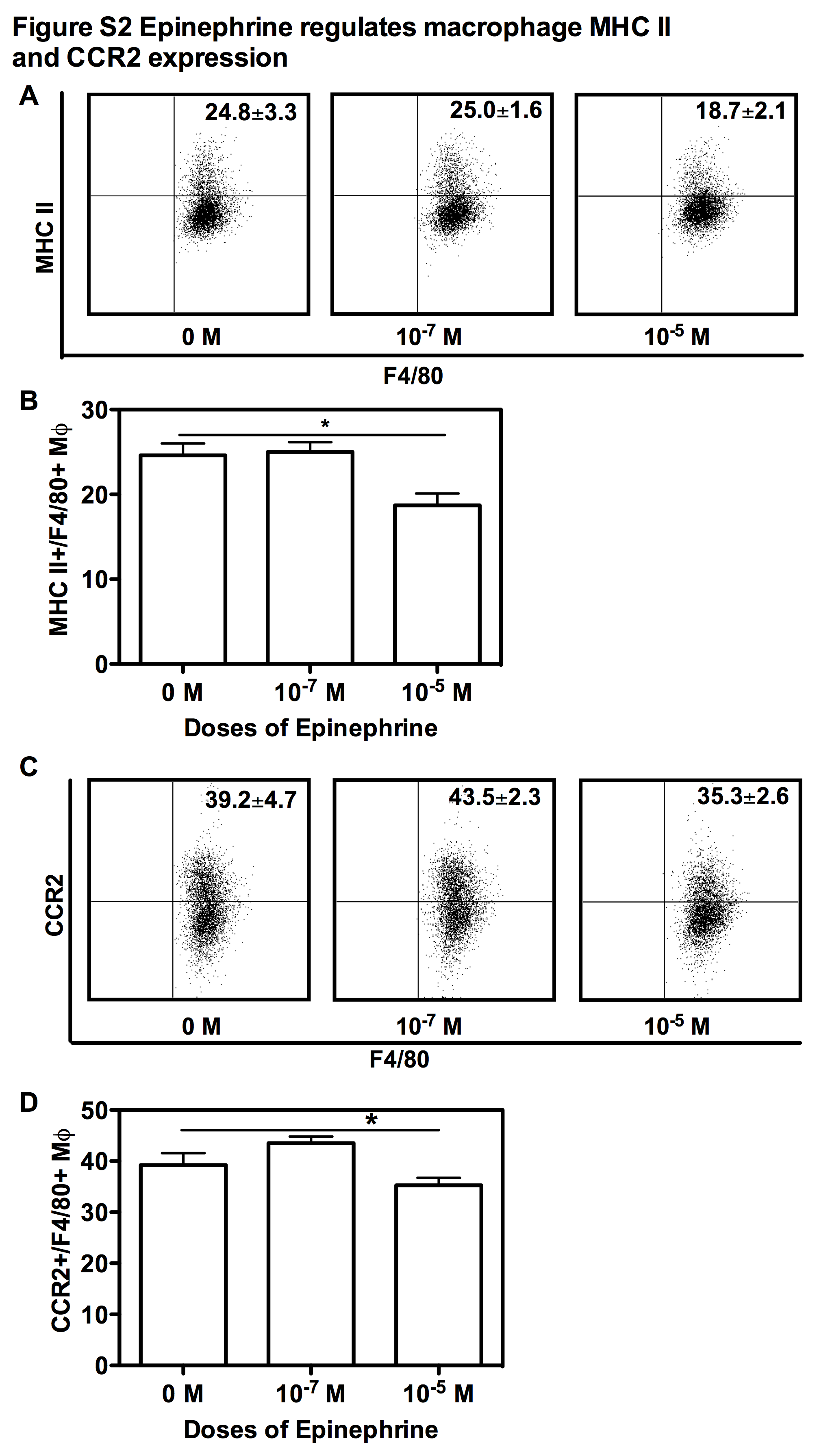

Supplement: Figure S2 — Epinephrine regulates MHC II and CCR2 expression of BMM. Unfractionated BM cells were plated in a 24-well plate at 2 x 10^6^ cells/well and cultured for 7 days in hormone-deficient medium with murine M-CSF alone, or in varying concentrations of epinephrine (1 x 10^-7^ M or 1 x 10^-5^ M) added at day 0. At day 7, cells were collected and stained with Abs for CD11b, MHC II, CCR2 and F4/80. Representative dot plot data of the percentage of MHC II+/F4/80+ Mφ and CCR2+/F4/80+ Mφ are shown in (A) and (C), respectively. The graphic format data of the percentage of MHC II+/F4/80+ Mφ and CCR2+/F4/80+ Mφ are shown in (B) and (D), respectively. Data show mean ± SD of 4 independent experiments. Significant difference is indicated as * p<0.05, compared to untreated control. (TIF) [file pone.0069167.s002.tif]
